# Associations Between Urinary Phthalate Metabolites and Decreased Serum α-Klotho Level: A Cross-Sectional Study Among US Adults in Middle and Old Age

**DOI:** 10.3390/toxics12110817

**Published:** 2024-11-14

**Authors:** Yuyan Liu, Xiaoyu Zhao, Shuxian Ma, Yongfang Li

**Affiliations:** 1 Department of Clinical Epidemiology, The Fourth Affiliated Hospital of China Medical University, Shenyang 110031, China; yyliu@cmu.edu.cn; 2 Key Laboratory of Environmental Stress and Chronic Disease Control & Prevention, Ministry of Education, China Medical University, Shenyang 110122, China; zxy3439985434@163.com (X.Z.); msx001267@163.com (S.M.); 3 School of Public Health, China Medical University, Shenyang 110122, China; 4 The Key Laboratory of Liaoning Province on Toxic and Biological Effects of Arsenic, Shenyang 110122, China

**Keywords:** phthalates, Klotho, cross-sectional study, aging, NHANES

## Abstract

Phthalates are widely used chemicals with ubiquitous human exposure. Evidence indicated that phthalate exposure was associated with an increased risk of aging-related diseases. Klotho is a transmembrane protein with anti-aging functions, and its association with phthalates remains unknown. To find the association between phthalate exposure and serum α-Klotho, a cross-sectional study was performed in 4482 adults (40–79 years old) who completed the National Health and Nutrition Examination Survey (NHANES) (2007–2016). As shown in the results of multivariable linear regression analyses, mono(carboxynonyl) phthalate (MCNP) and mono-n-butyl phthalate (MBP) were inversely associated with α-Klotho, and the regression coefficients of MCNP and MBP were −1.14 (95% confidence interval (CI): −2.00, −0.27) and −0.08 (95% CI: −0.14, −0.02). Subgroup analyses based on the quartiles of each phthalate metabolite showed that both MCNP and MBP were only inversely associated with α-Klotho in the subgroups of the highest levels. For mono-isobutyl phthalate (MIBP), the inverse association with α-Klotho was only statistically significant in the subgroup of the lowest level, and the regression coefficient was −26.87 (95% CI: −52.53, −1.21). Our findings suggest that α-Klotho might be involved in the association of phthalate exposure with aging-related diseases. Future research investigating the causality between phthalates and α-Klotho and its underlying mechanisms is encouraged.

## 1. Introduction

Phthalates are a series of synthetic chemicals widely used in plastic industries to improve the durability and flexibility of various products, including building materials, clothing, cosmetics, pharmaceuticals, nutritional supplements, medical devices, food packaging, cleaning materials and insecticides [1]. It has been reported that in 2018, the total global production of phthalates was around 5.5 million tons [2]. Phthalates can easily be released into the living environment during the process of manufacturing, leading to ubiquitous human exposure. The original forms or metabolites of phthalates have frequently been detected in various biological matrices, such as serum, urine and breast milk [3,4,5]. Several dysfunctions/diseases, including those correlated with aging, have been found to be associated with phthalate exposure. For example, Lind et al. reported that among 1016 elderly people, serum biomarkers of phthalates were positively associated with obesity and diabetes, as well as carotid atherosclerosis [6,7,8]. In another study including 474 Chinese participants, di-(2-ethylhexyl) phthalate (DEHP) was found to be associated with increased blood pressure [9]. The positive associations of phthalates with obesity and insulin resistance were also reported in studies based on the data of the National Health and Nutrition Examination Survey (NHANES) [10,11].

The potential mechanisms underlying associations between phthalate exposure and aging-related diseases are not well disclosed so far, although some aging-related biomarkers have been identified to be associated with phthalate exposure. Both experimental and populational studies have reported that phthalate exposure could be positively associated with thiobarbituric acid-reactive substances (TBARSs), 8-hydroxy-2’-desoxyguanosine (8-OHdG) and malondialdehyde (MDA) [12,13]. All of these biomarkers are closely correlated with oxidative stress, which plays an important role in aging-related dysfunctions/diseases.

Klotho is a transmembrane protein and considered as a human aging-suppression molecule. Among the isoforms of Klotho, α-Klotho was the first discovered and attracts the most extensive attention [14]. In humans, α-Klotho is encoded by the KL gene, and is predominantly expressed in the kidney and brain [14,15,16,17]. Accumulating evidence has indicated that the anti-aging role of α-Klotho might be associated with its regulation effects on phosphate metabolism [14]. As a co-receptor of fibroblast growth factor 23 (FGF23), α-Klotho facilitates the work of FGF23 in decreasing the level of sodium-dependent phosphate transport protein 2A (NPT2A), and subsequently inhibiting phosphate reabsorption. Moreover, the antioxidant effect of α-Klotho can also contribute to its anti-aging role. It has been reported that the overexpression of α-Klotho can suppress the activity of reactive oxygen species (ROS)-sensitive apoptosis signal-regulating kinase 1 (ASK1) [18].

Epidemiological studies have disclosed that the serum α-Klotho level decreases with age [19,20,21], and inverse associations between serum α-Klotho level and aging-related diseases have also been reported in recent years. There is a cohort study with 2774 American older adults showing that a higher baseline serum α-Klotho level was associated with a lower incidence of hypertension [22]. In another cohort study of 2238 Korean participants, an inverse association between α-Klotho and metabolic syndrome was also disclosed [23]. A recent meta-analysis study also found that a lower α-Klotho level was associated with an increased risk of mortality in patients with chronic kidney diseases [24]. Moreover, based on the anti-aging effect of α-Klotho, experimental studies have reported that a treatment increasing α-Klotho levels exerted therapeutic roles in aging-related diseases, such as hypertension, chronic kidney disease and ischemic heart disease [25,26].

Given the anti-aging properties of α-Klotho, as well as the positive association between phthalate exposure and aging-related diseases, it would be of interest to elucidate whether the inverse association between phthalate exposure and decreased α-Klotho exists. Even though no epidemiology study has disclosed the associations between phthalates and α-Klotho so far, there might be some possible underlying pathophysiological mechanisms relevant to oxidative stress. It has been reported that long-term exposure to phthalates might disrupt the activities of antioxidant proteins, which probably contributes to the decreased α-Klotho level [27,28,29]. In addition, the epigenetic modulation effect of phthalates might also potentially contribute [30]. In accordance with evidence from experimental studies, phthalates could perhaps bind to histone tails, and thereafter interrupt DNA methylation and demethylation, as well as the condensation of chromatin at the promoter region of critical genes, such as the KL gene [31,32].

Therefore, all this abovementioned evidence motivated us to perform this cross-sectional study based on NHANES data, aiming to estimate associations between urinary phthalate metabolites and serum α-Klotho levels. We thought this study could be helpful in finding out if α-Klotho was a biomarker of aging-related diseases in susceptible people exposed to phthalates for long durations, and could provide new evidence for further clarifying the underlying pathophysiological mechanisms of phthalate exposure in aging-related diseases.

## 2. Materials and Methods

### 2.1. Study Population

**Figure 1:**
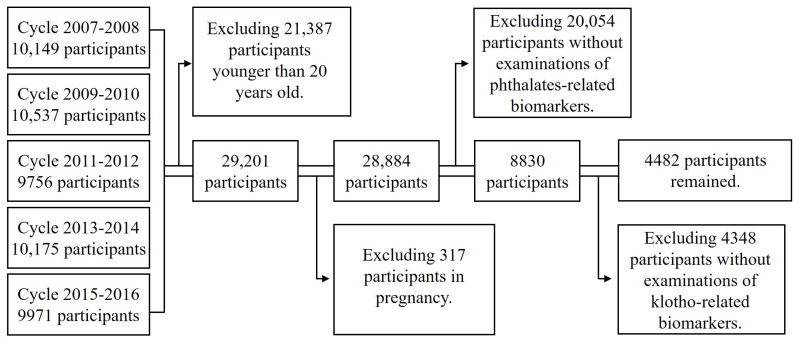
Flowchart of this cross-sectional study.

The NHANES program is a continuous, large-scale health investigation program in the United States, aiming to determine the risk factors of diseases, give evidence for standardizing health-related measurements and eventually direct the sound health policy (https://www.cdc.gov/nchs/nhanes/about_nhanes.htm, accessed on 10 November 2023). Since 1999, the program randomly selects 15 counties across the country each year and recruits participants of a representative sample size to join the survey, including questionnaire investigations, anthropometric examinations and laboratory tests. We performed this study by using the data from 5 survey cycles of NHANES from 2007 to 2016, considering that both α-Klotho and phthalate metabolites were examined in this timeframe. In brief, as shown in Figure 1, 50,588 participants in total were investigated in 5 cycles from 2007 to 2016, and 21,387 participants younger than 20 years old were first excluded. We further excluded those who were not eligible for the following analyses based on our exclusion criteria, including pregnant women and any participant with missing data of α-Klotho or phthalate-related examinations. We did not exclude participants with missing values of other variables apart from serum α-Klotho and urinary phthalates at the beginning of the analyses, while in subsequent multivariable regression models, only those without any missing values of α-Klotho, phthalates and relevant covariables were included. As a result, 4482 participants remained for the following analyses. The NHANES protocol was approved by the National Center for Health Statistics (NCHS) Institutional Review Board (https://www.cdc.gov/nchs/nhanes/irba98.htm, accessed on 10 November 2023), and participants recruited in the studies provided written consent.

### 2.2. Examinations of Urinary Phthalate Metabolites

The urinary chemical samples were collected at mobile examination centers (MECs) and stored frozen (−20 °C). High-performance liquid chromatography–electrospray ionization tandem mass spectrometry (HPLC-ESI-MS/MS) was used to detect phthalate metabolites in urine, i.e., mono(carboxynonyl) phthalate (MCNP), mono(carboxyoctyl) phthalate (MCOP), mono-2-ethyl-5-carboxypentyl phthalate (MECP), mono-n-butyl phthalate (MBP), mono-(3-carboxypropyl) phthalate (MC1), mono-cyclohexyl phthalate (MCP), mono-ethyl phthalate (MEP), mono-(2-ethyl-5-hydroxyhexyl) phthalate (MHHP), mono-(2-ethyl)-hexyl phthalate (MHP), mono-n-methyl phthalate (MNMP), mono-isononyl phthalate (MNP), mono-(2-ethyl-5-oxohexyl) phthalate (MOHP), mono-n-octyl phthalate (MOP), mono-benzyl phthalate (MZP), mono-isobutyl phthalate (MIBP), cyclohexane 1,2-dicarboxylic acid monohydroxy isononyl ester phthalate (MHNCP), mono-2-hydroxy-iso-butyl phthalate (MHIBP), cyclohexane-1,2-dicarboxylic acid-mono(carboxyoctyl) ester phthalate (MCOHP) and mono-3-hydroxy-n-butyl phthalate (MHBP). In addition, 4-methyl umbelliferone glucuronide was used to monitor deconjugation efficiency. This selective method allows for the rapid detection of the monoester metabolites of commonly used phthalate diesters in human urine with limits of detection (LODs) in the low ng/mL range. The levels below the LOD were substituted with the LOD divided by the square root of two (https://wwwn.cdc.gov/Nchs/Nhanes/2015-2016/PHTHTE_I.htm, accessed on 10 November 2023). As shown in Table 1, in this current study, the following analyses were limited to phthalate metabolites that were continuously examined from 2007 to 2016 to ensure that the findings could be representative with a sufficient sample size. Moreover, MHP and MNP were further excluded, considering high percentages of participants lower than the LOD.

### 2.3. Examinations of α-Klotho

Whole blood samples were received on dry ice and stored at −80 °C, and then predefined batches of samples were provided daily to the technicians for analysis. The determination of α-Klotho concentrations was performed during the period of 2019–2020 by a commercially available ELISA kit produced by IBL International, Japan (https://wwwn.cdc.gov/Nchs/Nhanes/2015-2016/SSKL_I.htm, accessed on 10 November 2023). In priority, an extensive validation of the IBL ELISA method for measurement of α-Klotho concentration in human samples was performed. The assay’s standard curves and the relative signals of the calibrator concentrations were consistently within the criteria specified by the manufacturer. The obtained assay sensitivity was calculated to be 4.33 pg/mL, while the manufacturer-claimed sensitivity was 6.15 pg/mL. Two samples with very high and high Klotho concentrations were used at different dilutions to evaluate the assay linearity. Plots of the expected vs. the obtained values demonstrated an excellent linearity in the assay measurement range. The intra-assay precision obtained on two recombinant Klotho samples and two human samples exhibited a coefficient of variation (CV) of 3.2% and 3.9% for the recombinant and 2.3% and 3.3% for the human samples. The same samples analyzed in duplicate over 4 different days showed an inter-assay CV of 2.8% and 3.5% for the recombinant samples and 3.8% and 3.4% for the human samples. The reference ranges were evaluated in 114 samples from apparently healthy donors and the α-Klotho levels ranged from 285.8 to 1638.6 pg/mL with a mean of 698.0 pg/mL. The Northwest Lipid Metabolism and Diabetes Research Laboratories, Division of Metabolism, Endocrinology, and Nutrition, University of Washington, performed examinations. All sample analyses were performed according to the manufacturer’s protocol and all the results were checked to meet the laboratory’s standardized criteria for acceptability prior to being released for reporting.

### 2.4. Measurements of Confounders

Demographic information was collected via questionnaire investigations, including age, sex, race (Mexican American, non-Hispanic white/black, others), education levels (less than high school, high school, college or above), current smoking status (yes/no), alcohol drinking situation (yes/no) and prevalence of hypertension and type 2 diabetes mellitus (T2DM). Anthropometric measurements including body weight, height and waist circumference (WC) were obtained, and the body mass index (BMI) was then calculated as weight (kg) divided by the square of the height (m). According to the currently used cutoff point of BMI in defining obesity in the American population, we defined the general obesity as BMI ≥ 30 kg/m^2^. The central obesity was defined as WC ≥ 102 cm in men and ≥88 cm in women. Based on measurements of blood pressure, as well as questionnaire information, we defined hypertension as SBP ≥ 140 mmHg, or DBP ≥ 90 mmHg, or the use of antihypertensive medication. Biochemical variables were also included as confounders, including urinary creatinine and counts of lymphocytes, segmented neutrophils and platelets. In addition, the systemic immune-inflammation index (SII) was also calculated (SII = platelet count × neutrophil count/lymphocyte count).

### 2.5. Statistical Analysis

Considering that the NHANES has a complex multistage probability cluster design, variance estimates computed using standard statistical software packages that assume simple random sampling are generally biased because they do not account for differential weighting and the correlation among sample persons within a cluster (https://wwwn.cdc.gov/nchs/nhanes/tutorials/VarianceEstimation.aspx, accessed on 10 November 2023). Therefore, as per the recommendation of NCHS, we performed all statistical analyses of variance estimates based on Taylor series linearization procedures, in which variables of strata and primary sampling units (PSUs) in the stage of NHANES sampling, as well as weights created for taking complex survey design into account, were included.

Considering that the continuous variables did not follow normal distributions, medians with 25th and 75th percentiles were shown. Categorical variables were presented as percentages. In addition, the basic characteristics were also shown separately in men and women, and sex differences were analyzed using the Wilcoxon rank sum test and Chi-square test, respectively, for continuous and categorical variables. The adjusted associations of phthalate metabolites with α-Klotho were estimated using multivariable linear regression analyses by running the procedures of SURVEYREG. Three regression models were included, i.e., Model 1 (adjusted for urinary creatinine), Model 2 (adjusted for sex, age and race in addition to Model 1) and Model 3 (adjusted for education levels, BMI, hypertension, T2DM, smoking and alcohol drinking in addition to Model 2). In each model, only participants without any missing values of α-Klotho, phthalates or specific covariables were included. Moreover, we further divided the participants into 4 subgroups based on the quartiles of phthalate metabolites, and then performed linear regression analyses in each subgroup of quartiles (from Q1 to Q4). Stratified analyses of the associations between phthalate metabolites and α-Klotho were also performed based on the following categorical variables: sex (men/women); age (younger or older than 60 years old); central obesity (yes/no); general obesity (yes/no); SII (divided using medians); hypertension (yes/no); T2DM (yes/no); smoking status (yes/no); and alcohol drinking (yes/no).

All of the above statistical analyses were completed using SAS 9.4 (SAS Institute, Inc., Cary, NC, USA), and the analyses of variance estimates were performed by running SAS survey procedures. A *p*-value < 0.05 was considered statistically significant.

## 3. Results

### 3.1. General Characteristics

The general characteristics of all enrolled participants are shown as medians (25th and 75th percentiles) and percentages in Table 2. Among 4482 participants, 2192 were men (48.9%), and the median of age was 57.0 years old. Non-Hispanic white participants accounted for 72.8%, and 83.0% of participants had education levels of high school or above, among whom 31.0% were college graduates. According to the obesity criteria in the United States, participants in the current study were generally in a situation of overweight/obesity (the medians of BMI and WC were 28.7 kg/m^2^ and 100 cm, respectively). The median of urinary creatinine was 103.0 mg/dL, and the median of SII value was 452.4. Among participants, the percentages of current smoking and alcohol drinking were 18.8% and 80.0%, respectively. The prevalences of hypertension and T2DM were 41.9% and 14.5%, respectively. The median of serum α-Klotho level was 804.4 pg/mL. Medians of MCNP, MCOP, MECP, MBP, MC1, MEP, MHHP, MOHP, MZP and MIBP were 2.0 ng/mL, 8.8 ng/mL, 14.1 ng/mL, 12.3 ng/mL, 1.9 ng/mL, 56.5 ng/mL, 9.2 ng/mL, 5.6 ng/mL, 4.4 ng/mL and 7.6 ng/mL, respectively. In addition, differences by sex in the characteristics are shown in Table S1. We found that among our participants, men were older than women (*p*-value = 0.028) and had a lower BMI (*p*-value < 0.001) but a larger WC (*p*-value < 0.001). In men, a higher T2DM prevalence (*p*-value = 0.004) and lower α-Klotho level (*p*-value < 0.001) were found. No sex difference in any phthalate biomarker was found.

### 3.2. Associations Between Urinary Phthalate Metabolites and Serum α-Klotho

By running multivariable linear regression models based on the Taylor series linearization method, the statistically significant association between urinary phthalate metabolites and α-Klotho was only shown for MCNP in Model 1 (adjusted for urinary creatinine) (Table 3). The regression coefficient of MCNP was −0.99 (95% CI: −1.85, −0.13), and the *p*-value was 0.025, meaning that the serum α-Klotho level decreased by 0.99 pg/mL per 1 ng/mL increase in the urinary MCNP level. Consistent results were also shown in Model 2, where only MCNP was inversely associated with α-Klotho, in which the coefficient was −0.95 (95% CI: −1.80, −0.10) and the *p*-value was 0.029. By further adjusting confounders in Model 3, both MCNP and MBP were found to be inversely associated with α-Klotho (*p*-values were 0.011 and 0.015 for MCNP and MBP, respectively). The regression coefficients of MCNP and MBP were −1.14 (95% CI: −2.00, −0.27) and −0.08 (95% CI: −0.14, −0.02), i.e., per 1 ng/mL increase in MCNP and MBP, the α-Klotho levels decreased by 1.14 and 0.08 pg/mL, respectively.

The overall participants were then further divided into four subgroups according to the quartiles of each phthalate metabolite, and adjusted linear regression analyses were performed in each subgroup. As shown in Table 4, both MCNP and MBP were only inversely associated with α-Klotho in the subgroups of the highest levels (Q4) (*p*-values for MCNP and MBP were 0.001 and 0.030, respectively). The regression coefficients for MCNP and MBP were −1.66 (95% CI: −2.58, −0.73) and −0.05 (95% CI: −0.09, −0.01), i.e., per 1 ng/mL increase in MCNP and MBP, the α-Klotho levels decreased by 1.66 and 0.05 pg/mL, respectively. However, for MIBP, the inverse association with α-Klotho was only statistically significant in the subgroup of the lowest level (Q1) (*p*-value = 0.040), and the regression coefficient was −26.87 (95% CI: −52.53, −1.21), meaning that the serum α-Klotho level decreased by 26.87 pg/mL per 1 ng/mL increase in the urinary MIBP level.

### 3.3. Findings of Stratified Analyses in Subgroups of Confounders

**Figure 2:**
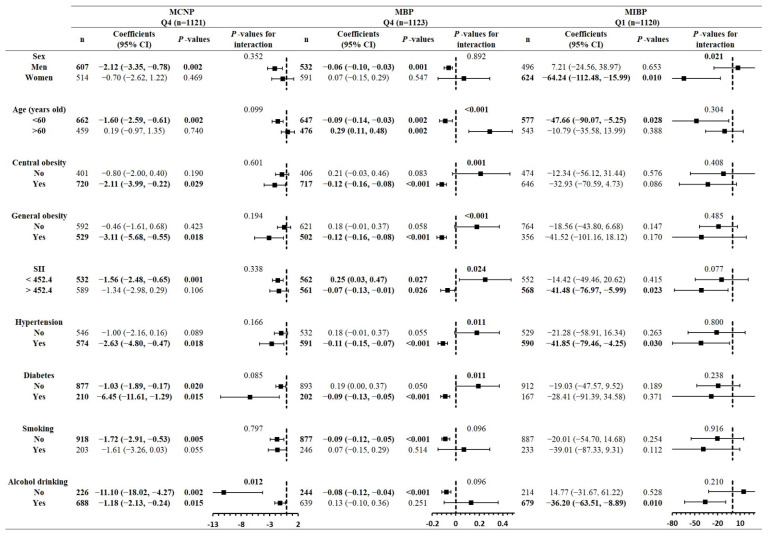
Stratified analyses of the interactive effects of confounders on associations of MCNP, MBP and MIBP with α-Klotho. The stratified analyses were performed in subgroups of the highest levels (Q4) of MCNP and MBP, as well as the lowest levels (Q1) of MIBP. Regression models are adjusted for urinary creatinine, sex, age, race, education levels, BMI, hypertension, T2DM, smoking and alcohol drinking. Values in bold are statistically significant. Abbreviations: MCNP: mono(carboxynonyl) phthalate; MBP: mono-n-butyl phthalate; MIBP: mono-isobutyl phthalate; 95% CI: 95% confidence interval.

To find the confounding effects of relevant risk factors on the associations between urinary phthalate metabolites and serum α-Klotho, stratified analyses were performed in subgroups of confounders. According to the results shown in Table 4, stratified analyses were only limited among participants in the highest quartiles of MCNP and MBP levels, respectively, as well as in the lowest quartile of MIBP levels, for whom statistically significant inverse associations with α-Klotho were found. As shown in Figure 2, for MCNP, inverse associations with α-Klotho were found in men, as well as in participants of a younger age; with central obesity, general obesity, lower SII values, hypertension and diabetes; and who did not smoke. Compared to alcohol drinkers, the inverse association was stronger in non-alcohol drinkers. The regression coefficients were −11.10 (95% CI: −18.02, −4.27) and −1.18 (−2.13, −0.24) for non-drinkers and drinkers, respectively, and a significant interaction was found (*p*-value for interaction = 0.012). For MBP, inverse associations with α-Klotho were found in men, as well as in participants of a younger age; with central obesity, general obesity, higher SII values, hypertension and diabetes; and who did not smoke or drink alcohol. The interactions by age, central obesity, general obesity, SII, hypertension and T2DM were found, and the *p*-values for these interactions were less than 0.001, 0.001, less than 0.001, 0.024, 0.011 and 0.011. However, opposite but significant results are shown in the subgroups of age and SII, and inverse associations were found in those younger than 60 years old and with a lower SII. For MIBP, inverse associations with α-Klotho were found in women, as well as in participants of a younger age, with higher SII values, with hypertension and who did not drink alcohol. A significant interaction by sex was found (*p*-value for interaction = 0.021), where the regression coefficients were 7.21 (95% CI: −24.56, −38.97) and −64.24 (−112.48, −15.99) for men and women, respectively.

## 4. Discussion

In this cross-sectional study, we found that urinary metabolites of phthalates (i.e., MCNP and MBP) were inversely associated with serum α-Klotho levels. By further performing analyses in the subgroups of the quartiles of phthalate metabolites, we found that inverse associations of MCNP and MBP with α-Klotho were only shown in participants with the highest exposure levels, while MIBP was found to be inversely associated with α-Klotho in those with the lowest exposure level. To the best of our knowledge, this is the first study disclosing the association between phthalate exposure and serum α-Klotho level in humans, and our findings could provide a new view in exploring the pathogenesis of the potential effects of phthalate exposure on aging-related dysfunctions/diseases.

In the NHANES, the examination of serum α-Klotho was not performed until the period of 2019 to 2020, and thus relationships between α-Klotho and environmental pollutant exposure have been reported in recent years, including p-dichlorobenzene, polycyclic aromatic hydrocarbons (PAHs), perfluorononanoic acid (PFNA), thiocyanate and heavy metals (lead and cadmium) [19,20,21,33,34]. However, the association between phthalate exposure and serum α-Klotho level has never been reported. In the current study, we included 19 urinary phthalate metabolites in the NHANES data (2007–2016), and 10 metabolites remained, which were continually examined with high rates of detection for our analyses. As far as we know, no study has included as many categories of phthalate metabolites as we did. Our findings imply that α-Klotho might play a potential role in the association between phthalate exposure and aging-related diseases, especially for MCNP and MBP.

Furthermore, by analyzing subgroups divided by quartiles of phthalate exposure levels, we found that the inverse associations of both MCNP and MBP with α-Klotho were only shown in participants with the highest exposure levels. Simultaneously, MIBP was found to be inversely associated with α-Klotho in those with the lowest exposure levels. Such findings suggest that nonlinearities perhaps exist in associations between various phthalate metabolites and α-Klotho, and dose–response relationships might be different among phthalate metabolites. In other words, for both MCNP and MBP, the effects of decreasing α-Klotho level might exist in high exposure doses, while for MIBP, a low exposure dose might be favorable for decreasing the α-Klotho level. We suppose that this such potential nonlinear association might be due to sampling errors and populational variations, and such findings still need to be identified in other populations. On the other hand, different pathophysiological mechanisms for decreasing the α-Klotho level might also exist among various phthalate metabolites, though these should be further clarified in both epidemiological and experimental studies.

The confounding effects of relevant risk factors on the associations between phthalate metabolites and α-Klotho were estimated using stratified analyses. As we found, for both MCNP and MBP, although no interaction by sex was found, statistically significant results were only shown in men. Zhu et al. has reported similar results, where among 1485 adults from the NHANES (2013–2016), the inverse association between urinary 2,5-dichlorophenols and α-Klotho was only found in men after adjustment [21]. However, for MIBP in our study, the inverse association with α-Klotho was found in women with a significant *p*-value for interaction. In contrast, with MCNP and MBP, people with the lowest urinary level of MIBP showed the inverse association with α-Klotho (Table 4), and thus the variance of participants might contribute to the differences in the interaction by sex. Nevertheless, in another study based on the NHANES (2007–2014), the urinary thiocyanate level was only inversely associated with α-Klotho in women (*p*-value for interaction = 0.003) [34]. In addition, we found that younger adults, as well as those with metabolic disorders (i.e., obesity, hypertension, T2DM) showed inverse associations of MCNP, MBP and MIBP with α-Klotho, implying that such people might be the susceptible population whose α-Klotho levels tend to be decreased due to phthalate exposure. Consistent findings have been reported for other pollutants. For example, both urinary thiocyanate and serum PFNA were found to be inversely associated with α-Klotho in people of less than 65 years old and of 40–59 years old, respectively [20,34]. Additionally, it was reported that the inverse association between thiocyanate and α-Klotho was only shown in those with obesity by performing stratified analyses [34]. Another result found in the current study is that positive associations between MBP and α-Klotho were shown in older participants, as well as those with lower SII values. Considering that phthalates are ensured harmful pollutants, we did not think that MBP could perform any protective effects by increasing α-Klotho levels. Such results of positive associations could perhaps be attributed to sampling errors and the limited sample size of the subgroups. Nevertheless, our findings about the interactive effects of confounders could still provide a direction for further research, and verifying the confounding effects of relevant risk factors on the associations between phthalates and α-Klotho would depend on longitudinal studies.

In our study, the HPLC-ESI-MS/MS detection method in examining urinary phthalate levels had detection thresholds for each phthalate biomarker. According to the NHANES’s instruction, the levels below the threshold detections were substituted with the LOD divided by the square root of two, so that we could be sure to obtain both reliable values of phthalate biomarkers and a sufficient sample size simultaneously. On the other hand, considering that the phthalate levels in our study were measured using urine samples, contact with some plastics during specimen acquisition, storage and sample analyses might interfere the concentrations of the phthalate biomarkers. In addition, prolonged exposure to room temperature during sample collection and/or transport could perhaps result in the degradation of the urine specimen and/or certain metabolites. However, strict procedures of quality control/assurance were performed in the NHANES so that reliable data could be obtained. For instance, urine specimens are required to be stored in the laboratory frozen at or below −40 °C prior to analysis. Prepared samples are kept at or below −20 °C until analysis and kept at 5 °C during SPE-HPLC-MS/MS analysis. Moreover, NCHS and contract consultants used a structured quality assurance evaluation during unscheduled visits to evaluate both the quality of the laboratory work and the quality-control procedures. Each laboratory staff person was observed for equipment operation, specimen collection and preparation. Formal retraining sessions were conducted annually to ensure that the required skill levels were maintained [35].

Caution should be paid in interpreting our findings. First, as a cross-sectional study, only associations between phthalates and α-Klotho, and not causalities, were estimated; longitudinal studies could be conducted to disclose their cause relationships. Second, findings in the current study were limited to only the US population, and associations between phthalate exposure and α-Klotho still need to be disclosed in other populations. Third, considering that the relevant data of geographical distribution could not be accessed, we did not know the exposure pathways of phthalates among populations. Therefore, we could not identify who might be at potential risk of phthalate exposure. Fourth, some other Klotho isoforms (i.e., β- and γ-Klotho) [14,16], as well as phthalate types (e.g., DEHP) were not examined in the NHANES, and as a result, associations among these biomarkers were not estimated in the current study. Nevertheless, considering that α-Klotho is the most representative Klotho isoform, and, simultaneously, ten continually examined metabolites of phthalates were included in our study, we thought our findings could well disclose the associations between phthalate exposure and decreased Klotho level. Last but not least, as we know, phthalate exposure could always be accompanied with other environmental pollutants, and relationships between phthalates and α-Klotho could probably be affected by other interactions. However, in the current study, the independent associations between phthalate exposure and α-Klotho were only considered, and thus relevant studies about co-exposure of multiple environmental pollutants should be continued. One of the strengths in this study is that our study was the first one disclosing the inverse associations between phthalate exposure and α-Klotho. Second, we included ten phthalate metabolites with high detection rates so that our findings could be more representative, and no study has included as many categories of phthalate metabolites as we did. Third, confounders were involved in the analyses, and the confounding effects on the associations between phthalate exposure and α-Klotho were found. Nevertheless, we could not include all confounders in the interaction analyses but only select some representative ones correlated with both phthalates and α-Klotho. Future studies focusing on phthalates and α-Klotho can take other variables with confounding effects into consideration.

## 5. Conclusions

In middle-aged and elderly adults in the United States, both the urinary levels of MCNP and MBP were inversely associated with serum α-Klotho level, especially in those with the highest quartile of exposure levels. For MIBP, the inverse association with α-Klotho was only shown in those with the lowest exposure levels. Our findings suggest that α-Klotho might be involved in the associations between phthalates and aging-related diseases, and future research could perhaps focus on disclosing the causality between phthalate exposure and decreased α-Klotho levels in various populations, as well as exploring the potential mechanisms underlying such associations.

## Figures and Tables

**Table 1:** The examinations of urinary phthalate metabolites in 5 cycles of the NHANES (2007–2016).

	n for Examination	LOD					n of LLODs	(%)
		2007–2008	2009–2010	2011–2012	2013–2014	2015–2016		
MCNP	13,502	0.500	0.200	0.200	0.200	0.200	190	1.4
MCOP	13,502	0.700	0.200	0.200	0.300	0.300	66	0.5
MECP	13,502	0.500	0.200	0.200	0.400	0.400	12	0.1
MBP	13,502	0.600	0.400	0.400	0.400	0.400	91	0.674
MC1	13,502	0.200	0.200	0.200	0.400	0.400	409	3.0
MCP	5353	0.603	0.462	N/G	N/G	N/G	5111	95.5
MEP	13,502	0.462	0.462	0.600	1.200	1.200	6	0.1
MHHP	13,502	0.700	0.200	0.200	0.400	0.400	31	0.2
MHP	13,502	1.100	0.500	0.500	0.800	0.800	1241	9.2
MNMP	7842	1.100	0.500	0.500	N/G	N/G	3502	44.7
MNP	13,502	1.232	0.770	0.500	0.900	0.900	3189	23.6
MOH	13,502	0.600	0.200	0.200	0.200	0.200	50	0.4
MOP	5353	1.848	0.840	N/G	N/G	N/G	5270	98.5
MZP	13,502	0.216	0.216	0.300	0.300	0.300	116	0.9
MIBP	13,502	0.300	0.200	0.200	0.800	0.800	95	0.7
MHNCP	8149	N/G	N/G	N/G	0.400	0.400	2871	35.2
HIBP	2975	N/G	N/G	N/G	N/G	0.400	112	3.8
MCOHP	2975	N/G	N/G	N/G	N/G	0.500	1192	40.1
MHBP	2975	N/G	N/G	N/G	N/G	0.500	648	21.8

Abbreviations: LOD: limit of detection; LLODs: levels below the LOD.

**Table 2:** General characteristics of all participants (n = 4482).

Characteristics	Medians (P25, P75) or n (%)
Men (%)	2192 (48.9)
Age (years)	57.0 (48.0, 66.0)
Race (%)	
Mexican American	709 (6.6)
Other Hispanic	502 (4.9)
Non-Hispanic white	1924 (72.8)
Non-Hispanic black	899 (9.2)
Others	448 (6.4)
Education level (%)	
Less than 9th grade	592 (6.4)
9–11th grade	665 (10.7)
High school graduation	1004 (22.1)
Some college graduation or AA degree	1215 (29.9)
College graduation and above	1004 (31.0)
BMI (kg/m^2^)	28.7 (25.2, 33.3)
WC (cm)	100 (90.1, 110.3)
Urinary creatinine (mg/dL)	103.0 (59.0, 156.0)
Count of lymphocytes (1000 cell/uL)	2.0 (1.6, 2.5)
Count of segmented neutrophils (1000 cell/uL)	3.9 (3.1, 5.0)
Count of platelets (1000 cell/uL)	232 (197, 276)
SII	452.4 (320.9, 643)
Current smoking (%)	882 (18.8)
Alcohol drinking (%)	2656 (80.0)
Hypertension (%)	2071 (41.9)
T2DM (%)	819 (14.5)
α-Klotho (pg/mL)	804.4 (658.4, 997.6)
MCNP (ng/mL)	2.0 (1.1, 4.1)
MCOP (ng/mL)	8.8 (3.9, 24.4)
MECP (ng/mL)	14.1 (7.0, 28.8)
MBP (ng/mL)	12.3 (5.8, 25.1)
MC1 (ng/mL)	1.9 (0.8, 4.2)
MEP (ng/mL)	56.5 (20.4, 185.1)
MHHP (ng/mL)	9.2 (4.3, 19.3)
MOH (ng/mL)	5.6 (2.7, 11.5)
MZP (ng/mL)	4.4 (1.9, 10.1)
MIBP (ng/mL)	7.6 (3.6, 14.3)

Continuous variables are shown as medians with 25th and 75th percentiles, and categorical variables are shown as numbers and percentages. Abbreviations: BMI: body mass index; WC: waist circumference; SII: systemic immune-inflammation index; T2DM: type 2 diabetes mellitus.

**Table 3:** Adjusted associations between urinary phthalate metabolites and serum α-Klotho (n = 4482).

	Model 1		Model 2		Model 3	
	Coefficients (95% CI)	*p*-Value	Coefficients (95% CI)	*p*-Value	Coefficients (95% CI)	*p*-Value
MCNP	**−0.99 (−1.85, −0.13)**	**0.025**	**−0.95 (−1.80, −0.10)**	**0.029**	**−1.14 (−2.00, −0.27)**	**0.011**
MCOP	0.08 (−0.12, 0.27)	0.438	0.07 (−0.12, 0.26)	0.483	−0.01 (−0.12, 0.09)	0.813
MECP	0.04 (−0.05, 0.14)	0.367	0.04 (−0.06, 0.14)	0.419	0.04 (−0.06, 0.14)	0.403
MBP	0.02 (−0.00, 0.04)	0.083	0.02 (−0.00, 0.04)	0.101	**−0.08 (−0.14, −0.02)**	**0.015**
MC1	0.07 (−0.26, 0.39)	0.685	0.05 (−0.28, 0.39)	0.747	−0.06 (−0.28, 0.17)	0.623
MEP	0.00 (−0.02, 0.01)	0.622	0.00 (−0.02, 0.01)	0.583	−0.01 (−0.01, 0.01)	0.846
MHHP	0.06 (−0.13, 0.25)	0.509	0.06 (−0.13, 0.25)	0.537	0.07 (−0.12, 0.26)	0.494
MOH	0.13 (−0.23, 0.50)	0.469	0.13 (−0.24, 0.50)	0.494	0.15 (−0.23, 0.52)	0.438
MZP	0.39 (−0.31, 1.09)	0.266	0.25 (−0.40, 0.90)	0.451	0.36 (−0.40, 0.12)	0.347
MIBP	0.43 (−0.34, 1.21)	0.271	0.37 (−0.35, 1.10)	0.310	0.28 (−0.50, 1.06)	0.473

Multivariable linear regression analyses were performed, and regression coefficients are shown. Model 1 is adjusted for urinary creatinine; Model 2 is adjusted for sex, age and race in addition to Model 1; Model 3 is adjusted for education levels, BMI, hypertension, T2DM, smoking and alcohol drinking in addition to Model 2. Values in bold are statistically significant. Abbreviations: 95% CI: 95% confidence interval.

**Table 4:** Adjusted associations between urinary phthalate metabolites and serum α-Klotho in subgroups divided using quartiles of phthalate metabolites (n = 4482).

	Q1		Q2		Q3		Q4	
	Coefficients (95% CI)	*p*-Value	Coefficients (95% CI)	*p*-Value	Coefficients (95% CI)	*p*-Value	Coefficients (95% CI)	*p*-Value
MCNP	−47.26 (−177.84, 83.31)	0.473	81.35 (−70.87, 233.58)	0.291	19.43 (−20, 58.85)	0.330	**−1.66** **(−2.58, −0.73)**	**0.001**
MCOP	−29.08 (−58.97, 0.81)	0.056	13.23 (−8.81, 35.28)	0.236	0.45 (−7.16, 8.06)	0.907	−0.03 (−0.14, 0.07)	0.532
MECP	7.46 (−9.39, 24.31)	0.381	7.58 (−7.14, 22.30)	0.309	3.46 (−3.43, 10.35)	0.321	0.05 (−0.05, 0.15)	0.303
MBP	−8.54 (−22.91, 5.84)	0.241	4.54 (−16.64, 25.72)	0.671	4.00 (−4.61, 12.61)	0.358	**−0.05** **(−0.09, −0.01)**	**0.030**
MC1	−130.44 (−294.62, 33.73)	0.118	62.26 (−32.86, 157.37)	0.196	−0.41 (−64.35, 63.54)	0.990	−0.06 (−0.26, 1.43)	0.561
MEP	−1.32 (−6.62, 3.98)	0.621	−4.13 (−9.21, 0.95)	0.110	−0.30 (−0.90, 0.29)	0.311	−0.01 (−0.03, 0.01)	0.234
MHHP	17.81 (−12.06, 47.69)	0.239	3.32 (−15.52, 22.17)	0.727	−2.47 (−13.45, 8.51)	0.656	0.07 (−0.12, 0.26)	0.487
MOH	47.52 (−20.04, 115.08)	0.165	24.12 (−7.05, 55.29)	0.127	−7.84 (−25.04, 9.36)	0.367	0.17 (−0.22, 0.55)	0.387
MZP	15.79 (−68.17, 99.75)	0.709	58.39 (−16.31, 133.09)	0.124	−9.60 (−26.22, 7.03)	0.254	0.51 (−0.30, 1.31)	0.214
MIBP	**−26.87** **(−52.53, −1.21)**	**0.040**	−19.3 (−48.77, 10.17)	0.196	4.37 (−12.28, 21.02)	0.603	0.32 (−0.43, 1.07)	0.404

Multivariable linear regression analyses were performed, and regression coefficients are shown. The regression models are adjusted for urinary creatinine, sex, age, race, education levels, BMI, hypertension, T2DM, smoking and alcohol drinking. Values in bold are statistically significant. Abbreviations: 95% CI: 95% confidence interval.

## Data Availability

The National Health and Nutrition Examination Survey data are publicly available at https://www.cdc.gov/nchs/nhanes/, accessed on 10 November 2023.

## Supplementary Materials

The following supporting information can be downloaded at https://www.mdpi.com/article/10.3390/toxics12110817/s1. Table S1: General characteristics of all participants divided by sex (n = 4482).
