# Supplementary material for: Associations Between Urinary Phthalate Metabolites and Decreased Serum α-Klotho Level: A Cross-Sectional Study Among US Adults in Middle and Old Age
Source: Toxics. 2024 Nov 14;12(11):817. doi: 10.3390/toxics12110817 (PMC11598463; doi:10.3390/toxics12110817)
Supplement: Supplementary file 1 [file toxics-12-00817-s001.zip › toxics-3295554-supplementary.pdf]

Associations between urinary phthalates metabolites and de-creased serum α-Klotho level: A cross-sectional study among US adults in middle and old age

Supplementary Table: Table S1

Table S1. General characteristics of all participants divided by sex (n = 4,482)

Characteristics	Medians (P25, P75) or n (%)		*P*-value
	Men (2,192)	Women (2,290)	
Age (years)	58.0 (49.0, 67.0)	57.0 (48.0, 66.0)	0.028
Race (%)			0.174
Mexican American	342 (15.6)	367 (16.3)	
Other Hispanic	227 (10.4)	275 (12.0)	
Non-Hispanic White	978 (44.6)	946 (41.3)	
Non-Hispanic Black	428 (19.5)	471 (20.6)	
Others	217 (9.9)	231 (10.1)	
Education level (%)			0.026
Less than 9th grade	303 (13.8)	289 (12.6)	
9-11th grade	332 (15.2)	333 (14.6)	
High school graduation	486 (22.2)	518 (22.6)	
Some college graduation or AA degree	551 (25.1)	664 (29.0)	
College graduation above	520 (23.7)	484 (21.2)	
BMI (kg/m^2)	28.5 (25.5, 32.2)	29.2 (24.7, 34.3)	<0.001
WC (cm)	102.1 (93.5, 112.0)	97.3 (86.4, 108.3)	<0.001
Urinary creatinine (mg/dL)	124.0 (78.0, 175.0)	84.0 (48.0, 133.0)	<0.001
Counts of lymphocytes (1000 cell/uL)	2.0 (1.6, 2.4)	2.0 (1.7, 2.5)	<0.001
Counts of segmented neutrophils (1000 cell/uL)	4.0 (3.1, 5.0)	3.9 (3.0, 5.0)	0.055
Counts of platelets (1000 cell/uL)	220 (186, 259)	245 (209, 293)	<0.001
SII	444.4 (318.2, 628.5)	462.1 (329.0, 658.5)	0.515
Current smoking (%)	480 (22.1)	402 (18.5)	0.003
Alcohol drinking (%)	1,424 (74.5)	1,232 (74.0)	0.722
Hypertension (%)	1,026 (46.9)	1,045 (45.6)	0.406
T2DM (%)	436 (20.6)	383 (17.2)	0.004
α-Klotho (pg/mL)	777.6 (645.0, 968.7)	832.3 (672.6, 1027.2)	<0.001
MCNP (ng/mL)	2.4 (1.2, 4.4)	1.8 (0.9, 3.7)	0.323
MCOP (ng/mL)	9.5 (4.1, 26.2)	8.3 (3.6, 23.0)	0.149
MECP (ng/mL)	15.1 (7.7, 30.5)	13.1 (6.4, 27.2)	0.064

MBP (ng/mL)	12.4 (6.0, 24.6)	12.3 (5.6, 25.7)	0.242
MC1 (ng/mL)	2.3 (1.0, 4.7)	1.6 (0.7, 3.8)	0.247
MEP (ng/mL)	55.5 (19.8, 186.6)	57.4 (21.2, 179.6)	0.079
MHHP (ng/mL)	10.2 (4.9, 20.8)	8.2 (3.8, 17.8)	0.036
MOH (ng/mL)	6.0 (3.1, 12.0)	5.2 (2.4, 10.9)	0.055
MZP (ng/mL)	4.8 (2.1, 10.4)	4.0 (1.7, 9.7)	0.128
MIBP (ng/mL)	7.9 (3.8, 14.3)	7.2 (3.3, 14.2)	0.507

Continuous variables are shown as medians with 25th and 75th percentiles, and categorical variables are shown as numbers and percentages. **Abbreviations:** BMI: body mass index; WC: waist circumference; SII: systemic immune-inflammation index; T2DM: type 2 diabetes mellitus.
